# HtpG Is a Metal-Dependent Chaperone Which Assists the DnaK/DnaJ/GrpE Chaperone System of Mycobacterium tuberculosis via Direct Association with DnaJ2

**DOI:** 10.1128/spectrum.00312-23

**Published:** 2023-04-06

**Authors:** Nikita Mangla, Ramandeep Singh, Nisheeth Agarwal

**Affiliations:** a Translational Health Science and Technology Institute, NCR Biotech Science Cluster, Faridabad, Haryana, India; b Jawaharlal Nehru University, New Delhi, Delhi, India; Johns Hopkins University School of Medicine

**Keywords:** *Mycobacterium tuberculosis*, proteostasis, heat shock proteins, chaperones, HSP90, HtpG, DnaK, DnaJ2, Clp protease

## Abstract

Protein folding is a crucial process in maintaining protein homeostasis, also known as proteostasis, in the cell. The requirement for the assistance of molecular chaperones in the appropriate folding of several proteins has already called into question the previously held view of spontaneous protein folding. These chaperones are highly ubiquitous cellular proteins, which not only help in mediating the proper folding of other nascent polypeptides but are also involved in refolding of the misfolded or the aggregated proteins. Hsp90 family proteins such as high-temperature protein G (HtpG) are abundant and ubiquitously expressed in both eukaryotic and prokaryotic cells. Although HtpG is known as an ATP-dependent chaperone protein in most organisms, function of this protein remains obscured in mycobacterial pathogens. Here, we aim to investigate significance of HtpG as a chaperone in the physiology of Mycobacterium tuberculosis. We report that M. tuberculosis HtpG (mHtpG) is a metal-dependent ATPase which exhibits chaperonin activity towards denatured proteins in coordination with the DnaK/DnaJ/GrpE chaperone system via direct association with DnaJ2. Increased expression of DnaJ1, DnaJ2, ClpX, and ClpC1 in a Δ*htpG* mutant strain further suggests cooperativity of mHtpG with various chaperones and proteostasis machinery in M. tuberculosis.

**IMPORTANCE** M. tuberculosis is exposed to variety of extracellular stressful conditions and has evolved mechanisms to endure and adapt to the adverse conditions for survival. mHtpG, despite being dispensable for M. tuberculosis growth under *in vitro* conditions, exhibits a strong and direct association with DnaJ2 cochaperone and assists the mycobacterial DnaK/DnaJ/GrpE (KJE) chaperone system. These findings suggest the potential role of mHtpG in stress management of the pathogen. Mycobacterial chaperones are responsible for folding of nascent protein as well as reactivation of protein aggregates. M. tuberculosis shows differential adaptive response subject to the availability of mHtpG. While its presence facilitates improved protein refolding via stimulation of the KJE chaperone activity, in the absence of mHtpG, M. tuberculosis enhances expression of DnaJ1/J2 cochaperones as well as Clp protease machinery for maintenance of proteostasis. Overall, this study provides a framework for future investigation to better decipher the mycobacterial proteostasis network in the light of stress adaptability and/or survival.

## INTRODUCTION

Aging, various physiological stresses, and diseases are known to trigger misfolding or aggregation of key cellular proteins along with alterations in their transcription and translation profiles. Distinct from other pathogens, Mycobacterium tuberculosis, the etiologic agent of tuberculosis (TB), encounters a vast number of unfavorable and stressful conditions within the host macrophages or granulomas. In the past, attempts have been made to comprehend the adaptive machinery of M. tuberculosis under stresses such as heat, hypoxia, acidic pH, oxidative stress, iron depletion, starvation, etc. Remarkably, M. tuberculosis has coevolved through the millennia and responds to these stresses with genome-wide transcriptomic and large-scale metabolomic changes, involving a conserved set of genes associated with protein homeostasis (also known as proteostasis).

Proteostasis is indispensable for survival of all living cells (1). Molecular chaperones, as prime coordinators of proteostasis, ensure the precise control of biogenesis, three-dimensional folding and maintenance of proteins in a functionally active state, along with their cellular turnover and degradation (2). Chaperones are ubiquitous proteins that are required at both cotranslational and posttranslational levels of protein biogenesis (3). They safeguard and preserve proteome integrity by enhancing the thermodynamically stable folding of nascent polypeptides, besides preventing the accumulation of potentially toxic, misfolded protein aggregates by refolding them (4). The evolutionarily conserved molecular chaperones, belonging to the heat shock protein (Hsp) family are well known for their role in proteostasis, both in eukaryotes and prokaryotes (1). Some of these Hsps are constitutively expressed, while others are induced in response to stresses such as hyperthermia or hypoxia and together with other cochaperones rescue aggregated or misfolded proteins, thus regulating cell growth and survival (5, 6). Annotated by molecular weight, several major families of Hsps such as Hsp100, Hsp90, Hsp70, Hsp60, and Hsp40 and small Hsps, are known (7, 8).

The highly conserved Hsp90 family proteins were initially identified in *Drosophila* spp. (9); however, their homologs are abundant and ubiquitously expressed among the species of higher eukaryotes to lower prokaryotes (1). Of note, HtpG, the bacterial homologue of eukaryotic HSP90, plays a key role in regulating several cellular processes, under both normal homeostasis and stress conditions (10–27). Nevertheless, the prokaryotic and eukaryotic Hsp90 homologs differ significantly in their stoichiometry, mode of action, and regulation (28–35).

HtpG comprises three distinct domains, *viz.*, an amino-terminal domain involved in ATP binding, the central domain that mediates ATP hydrolysis and binding to client proteins, and the carboxy-terminal domain, which is critical for dimerization. It cycles between an open and closed structural conformation, depending upon the absence or presence of ATP (36). In Escherichia coli, the heat shock response of HtpG is regulated by the *rpoH* gene encoding sigma factor-32 (37, 38). Other known moonlighting functions of HtpG include facilitation of *de novo* protein folding during stress, synthesis of genotoxin colibactin (secondary metabolite), and yersiniabactin, a siderophore (39).

HtpG helps in maintaining the integrity of a wide variety of client proteins, often in conjunction with diverse cofactors, which regulate its ATPase activity and conformational dynamics. For example, HtpG of E. coli (Ec-HtpG) functions in concurrence with DnaK (Hsp70) and its cochaperones such as DnaJ (an Hsp40), as well as a nucleotide exchange factor, GrpE. Interestingly, Ec-HtpG interacts with DnaK at its nucleotide binding domain, which also involves the DnaJ binding region (40). Posttranslational modifications such as phosphorylation and acetylation also regulate this system. Unlike prokaryotic HtpG, for which only a few client proteins such as ribosomal protein L2 (41), DNA-replication initiator DnaA (42), and cell-division protein FtsZ (43) are known till date, eukaryotic HSP90 normalizes hundreds of client proteins involved in diverse cellular processes, such as protein folding, stress regulation, DNA repair, neuronal signaling, immune response, metabolism, cellular growth and development, etc.

In the hostile niche within the host, dysregulation of protein metabolism may fatally impede critical cellular pathways, thus making proteostasis one of the primary and vital arsenals of M. tuberculosis’s adaptive response. Indeed, this may be the reason why the TB pathogen has preserved various proteostatic components, including an array of chaperons and proteolytic machinery, through the course of evolution. While a considerable amount of information is available about M. tuberculosis chaperons and unfoldases such as DnaK, GroEL/ES, ClpB, ClpX, ClpC1, etc., we have yet to understand the role of M. tuberculosis HtpG encoded by *Rv2299*c (mHtpG), which is primarily restricted to the pathogenic mycobacteria. In this study we report that mHtpG is a metal-dependent ATPase which exhibits chaperonin activity towards denatured proteins in coordination with the DnaK/DnaJ/GrpE (KJE) chaperone system. Notably, the association of mHtpG with the KJE system involves direct interaction with DnaJ2. Quantitative mass spectrometry analysis of differentially expressed proteins in Δ*htpG* further suggests cooperativity of HtpG with various chaperones and protease machinery for the maintenance of M. tuberculosis proteome.

## RESULTS

### mHtpG is a highly conserved heat shock protein.

**FIG 1:**
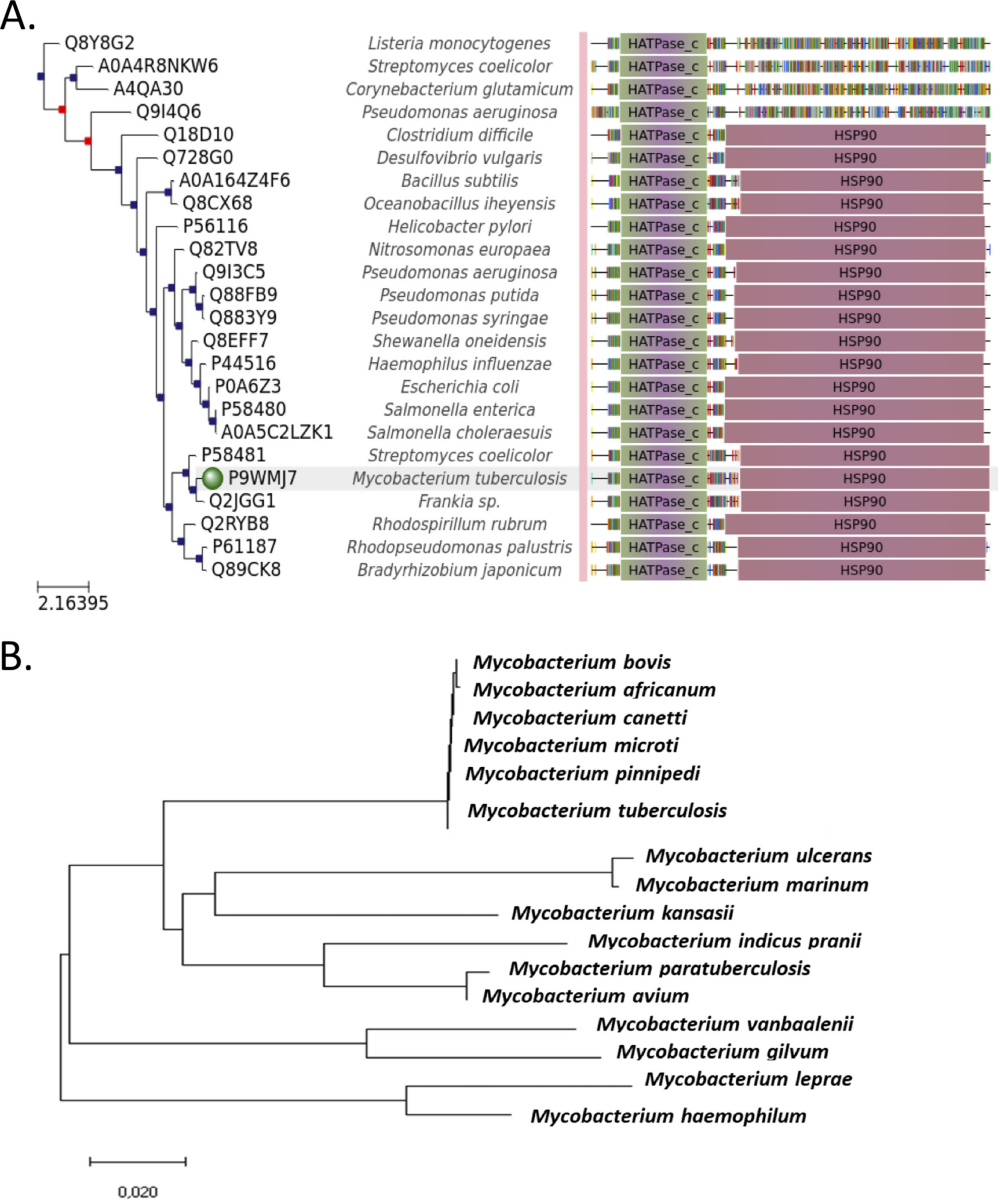
Phylogenetic analysis of mHtpG. (A) Phylogenetic tree analysis of mHtpG. The phylogenetic tree was generated for mHtpG by using the phylomeDB database (http://phylomedb.org/). The interactive tree can be accessed by the following link: http://phylomedb.org/phylome_328?id=3&seqid=Phy001DWYA&phyid=328&method=LG&tree_features=motifs,lineage,support,best_name,spname&treeid=&sid=&seedid=001DWYA&snodes=&isPopUp=False&isExport=False&isPrincipal=True&snodes=#search_in_tree_box. The protein sequence of different bacterial species was retrieved from NCBI (https://www.ncbi.nlm.nih.gov/). (B) Evolutionary relationship of mHtpG with its counterpart from different mycobacterial species. Evolutionary analyses were conducted using MEGA X software (https://www.megasoftware.net/). The evolutionary relationship was inferred using the neighbor-joining method. The optimal tree with the sum of branch length 0.65537958 is shown. The evolutionary distances were computed using the Poisson correction method and are presented as the number of amino acid substitutions per site. This analysis involved 17 amino acid sequences. All ambiguous positions were removed for each sequence pair (pairwise deletion option). There were 674 positions in the final data set. Scales are shown in panels A and B for estimation of branch lengths between the nodes.

Phylogenetic analysis depicts the evolutionary divergence of the HtpG protein sequence among different bacterial species (Fig. 1A). Apart from this, the phylogenetic tree reveals close association of mHtpG with its counterpart from other M. tuberculosis complex bacteria such as M. bovis, M. africanum, M. canettii, M. microti, and M. pinnipedii, as assessed by the evolutionary distance between these nodes (Fig. 1B). This is further corroborated by the multiple sequence alignment of HtpG proteins from M. tuberculosis complex bacteria as well as from other mycobacterial species (see Fig. S1 at https://thsti.in/Supplementary%20Information_Mangla%20et%20al%20Final-R1.pdf). Importantly, mHtpG was conserved in the pathogenic M. tuberculosis complex group, but no homolog is present in the avirulent M. smegmatis. From the phylogenetic tree analysis, we also made a striking observation which reveals that the HtpG of slow-growing M. ulcerans is closely associated with that of fast-growing M. marinum, suggesting that the two proteins are evolved together from a common ancestor (Fig. 1B; see Fig. S1 at the URL mentioned above). Despite differences in the rate of proliferation, both these organisms require a relatively lower temperature of 29 to 33°C for optimum growth. The above-described result thus suggests that these bacteria have acquired parallel changes in their respective HtpG proteins during the course of evolution. Indeed, sequence alignment reveals 99% identity between the two proteins (see Fig. S1 at the URL mentioned above), further suggesting their common ancestral origin during evolution.

### mHtpG exhibits metal-dependent ATPase activity.

**FIG 2:**
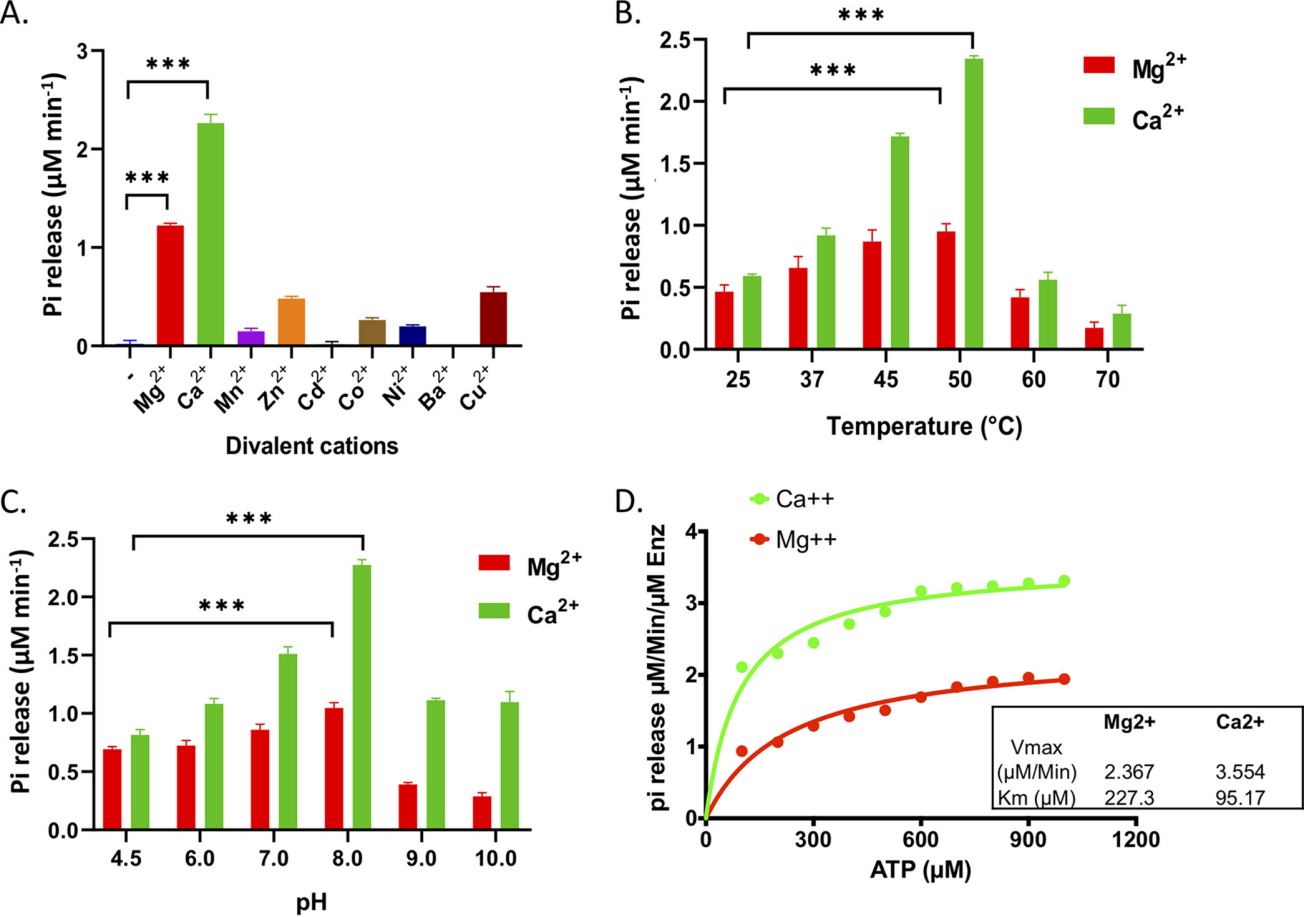
Analysis of ATPase activity of mHtpG. (A) Impact of different divalent cations on the ATPase activity. Shown is the effect of different metal ions such as Mg^++^, Ca^++^, Mn^++^, Zn^++^, Cd^++^, Co^++^, Ni^++^, Ba^++^, and Cu^++^ on the ATP hydrolysis activity of mHtpG. The assay was performed by using 1 μM mHtpG for 30 min, and the released Pi was estimated using a malachite green assay, as described in Materials and Methods. (B and C) Effect of temperature (B) and pH (C) on ATPase activity of mHtpG. Reactions were performed at various temperatures, such as 25°C, 37°C, 45°C, 50°C, 60°C, and 70°C, at pH 7.5 of the reaction buffer in panel B, and at different pHs of the reaction buffer, such as 4.5, 6.0, 7.0, 8.0, 9.0, and 10.0 at 50°C in panel C for 30 min, and the released Pi was estimated as described above. (D) Comparative analysis of the effect of Mg^++^ and Ca^++^ on kinetics of the ATPase activity of mHtpG. Reactions were performed with 1 μM mHtpG using different concentrations of ATP varying from 100 μM to 1,000 μM in the presence of Mg^++^ and Ca^++^ divalent cations. Kinetic parameters such as *K_m_* and *V*_max_ were determined using GraphPad Prism 8.0 software (https://www.graphpad.com/scientific-software/prism/). All the reactions in panels A to D were performed in triplicate, and the mean ± standard deviation (SD) values are shown. Asterisks represent the level of significance, as determined by Student’s *t* test. ***, *P* ≤ 0.0005.

Sequence analysis of mHtpG reveals the presence of the ATPase domain in the N-terminal half and the Hsp90 domain in the middle and the C-terminal half (see Fig. S2A at https://thsti.in/Supplementary%20Information_Mangla%20et%20al%20Final-R1.pdf). In order to determine the catalytic efficiency of mHtpG, its ATP hydrolyzing activity was analyzed by using the malachite green assay, as described in Materials and Methods. The glutathione *S*-transferase (GST) tag from the purified GST-mHtpG was removed prior to performing any assay, and the untagged protein was purified by size exclusion chromatography and analyzed by SDS-PAGE, which confirms the removal of GST tag (see Fig. S2B at the URL mentioned above). To identify the optimum condition for ATP hydrolysis by mHtpG, various conditions, including different divalent cations, reaction temperatures, and the pH of the reaction buffer, were tested. In order to check for the specific requirement of metal ions, the assay was performed in the absence and the presence of various divalent cations. Our results show that ATPase activity of mHtpG is strictly dependent on divalent cations such as Mg^++^ and Ca^++^, as no activity is found in the absence or the presence of any other divalent cations (Fig. 2A). Remarkably, the presence of Ca^++^ significantly enhances the ATPase activity by ~2-fold compared with the enzyme’s activity in the presence of Mg^++^ (Fig. 2A). Furthermore, we observe that the ATP hydrolysis activity of mHtpG varies with the temperature and pH of the buffer. The reaction is most favored when performed at 50°C and pH 8.0 with either the Mg^++^ or Ca^++^ in the reaction buffer (Fig. 2B and C). Analysis of kinetic parameters at different concentrations of the substrate further reveals that mHtpG exhibits ATPase activity with a *K_m_* of ~227.3 μM and *V*_max_ of ~2.367 μM/min in the presence of Mg^++^. Importantly, the affinity of mHtpG with ATP and the rate of reaction are significantly enhanced in the presence of Ca^++^, with a *K_m_* of ~95.17 μM and *V*_max_ of ~3.554 μM/min (Fig. 2D).

### mHtpG stimulates protein refolding by the KJE chaperone system.

**FIG 3:**
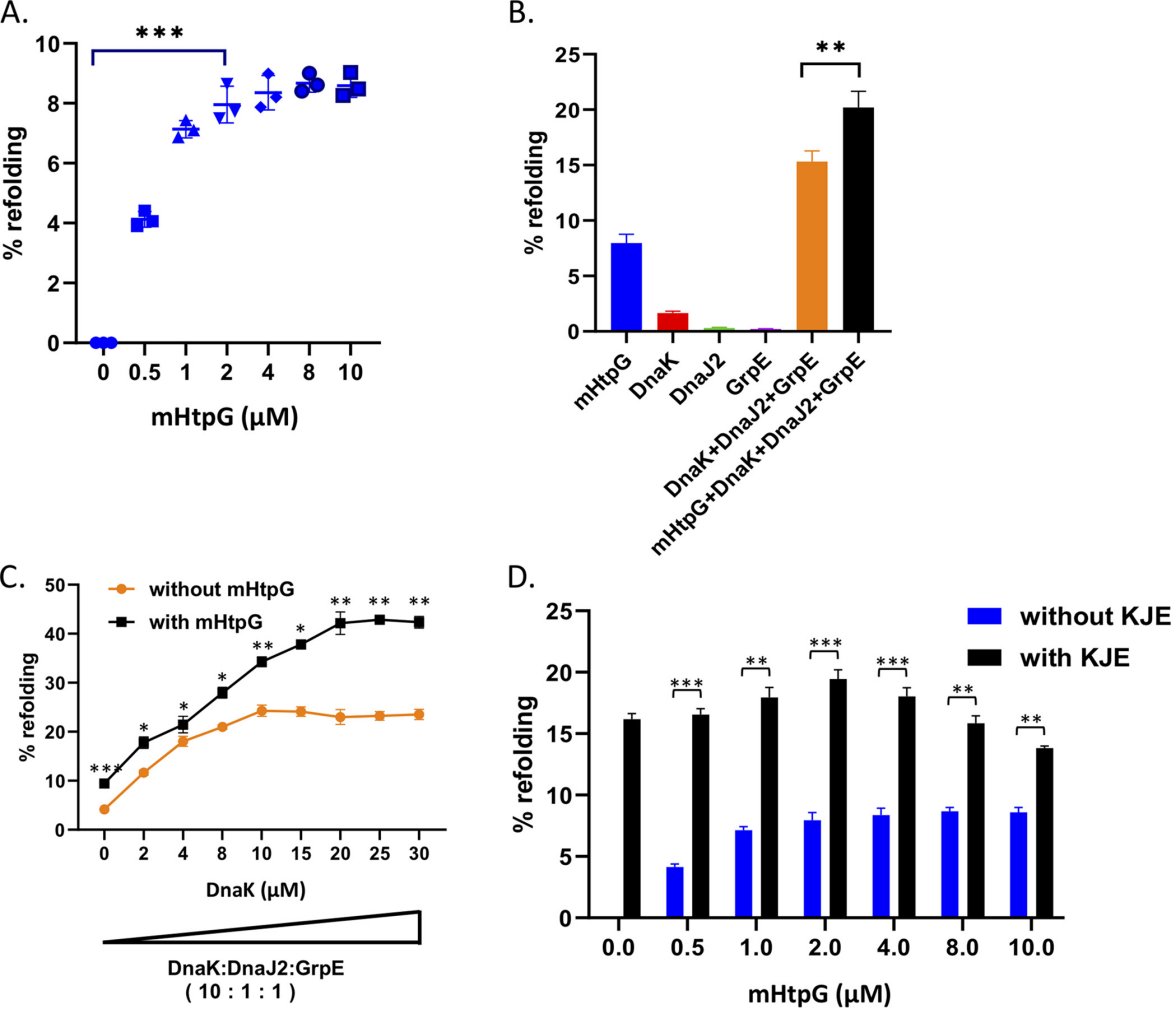
Analysis of refolding activity of mHtpG. (A) Dose-dependent effect of mHtpG on the refolding of denatured luciferase. The refolding activity of mHtpG was analyzed at different protein concentrations using 80 nM heat-denatured luciferase, as described in Materials and Methods. (B) Effect of mHtpG on luciferase refolding by the mycobacterial KJE chaperone. The refolding assay was performed with 2 μM mHtpG and DnaK/DnaJ2/GrpE (4 μM/400 nM/400 nM) proteins either alone or in combination to explore the synergistic effect of each of these components. (C) Dose-dependent effect of KJE on refolding in the absence and the presence of mHtpG. The refolding activity of the KJE chaperone system was examined using increasing concentrations of chaperones and constant mHtpG (2 μM). (D) Dose-dependent effect of mHtpG on refolding of denatured luciferase in the presence of the KJE chaperone system. The effect of the KJE chaperone system on the refolding activity of mHtpG was analyzed by using various concentrations of mHtpG and a fixed concentration (4 μM/400 nM/400 nM) of DnaK/DnaJ2/GrpE. All the reactions in (A to D) were performed in triplicate, and the mean ± SD values are shown. Asterisks represent the level of significance, as determined by Student’s *t* test. *, *P* < 0.05; **, *P* < 0.005; ***, *P* ≤ 0.0005.

Next, to assess the chaperonin activity of mHtpG, it was subjected to a protein refolding assay by using denatured firefly luciferase as a model substrate. The firefly luciferase was thermally denatured such that it remains soluble while losing most of its activity. Refolding of this protein was subsequently examined upon incubation with mHtpG. It was observed that in the presence of mHtpG, the activity of denatured luciferase is marginally restored to ~8% of the native protein’s activity (Fig. 3A). A dose-dependent increase in the refolding is seen with up to 2 μM mHtpG, whereas no further change is noticed by increasing the mHtpG beyond 2 μM (Fig. 3A). Since Ec-HtpG is known to influence the refolding activity of the DnaK/DnaJ/GrpE (collectively annotated as KJE) chaperone system (42, 44), we next examined whether a similar effect is imparted by mHtpG on mycobacterial KJE. The M. tuberculosis KJE chaperone system includes DnaK and its cochaperones DnaJ and GrpE. Although M. tuberculosis possesses two bacterial homologs of Hsp40, DnaJ1 and DnaJ2, only one of these two DnaJ proteins is essentially required in M. tuberculosis for its optimal cellular functions (45). Since the recombinant DnaJ1 of M. tuberculosis could not be purified from E. coli as a soluble protein, we utilized M. tuberculosis DnaJ2 to reconstitute the KJE system. As anticipated, the refolding requires all three components, as the absence of GrpE or DnaJ2 drastically affects the chaperonin activity of DnaK (Fig. 3B). The refolding activity of M. tuberculosis KJE is further improved from 15.3% to 20.2% upon addition of 2 μM mHtpG in the KJE complex containing 4 μM DnaK complexed with GrpE and DnaJ2 in 10:1:1 ratio (Fig. 3B).

Although DnaK exhibits strong affinity to the model substrate, which binds with DnaK within a few seconds of addition, folding of the substrate by the KJE system takes longer, as it involves multiple steps (46) and thus can be rate limiting. By varying the concentrations of DnaK in the KJE complex, it was observed that the refolding activity of DnaK comes to a plateau beyond 10 μM (Fig. 3C), which could be due to the formation of deadlock as reported for Ec-Hsp70 (44). These results further indicate that the M. tuberculosis KJE system may require additional factor(s) for productive folding of substrate. In order to examine whether mHtpG is one such accessory protein which helps M. tuberculosis KJE with protein folding, we titrated the level of DnaK in the KJE complex in the presence of mHtpG. Interestingly, we find that the addition of mHtpG stimulates the luciferase folding by KJE in a progressive manner, and there is a linear increment in the folding from 0 to 20 μM DnaK (Fig. 3C). Importantly, the addition of mHtpG leads to an ~100% increase in the refolding of luciferase by KJE comprising 20 μM DnaK (Fig. 3C). Notably, increasing concentrations of mHtpG at a fixed concentration of KJE does not have any major impact on refolding (Fig. 3D). Taken together, these results suggest primary involvement of M. tuberculosis KJE in the stimulation of folding of denatured substrate, whereas mHtpG seems to play an assisting role.

### ATP hydrolyzing activity of mHtpG is vital for its assistance to KJE.

**FIG 4:**
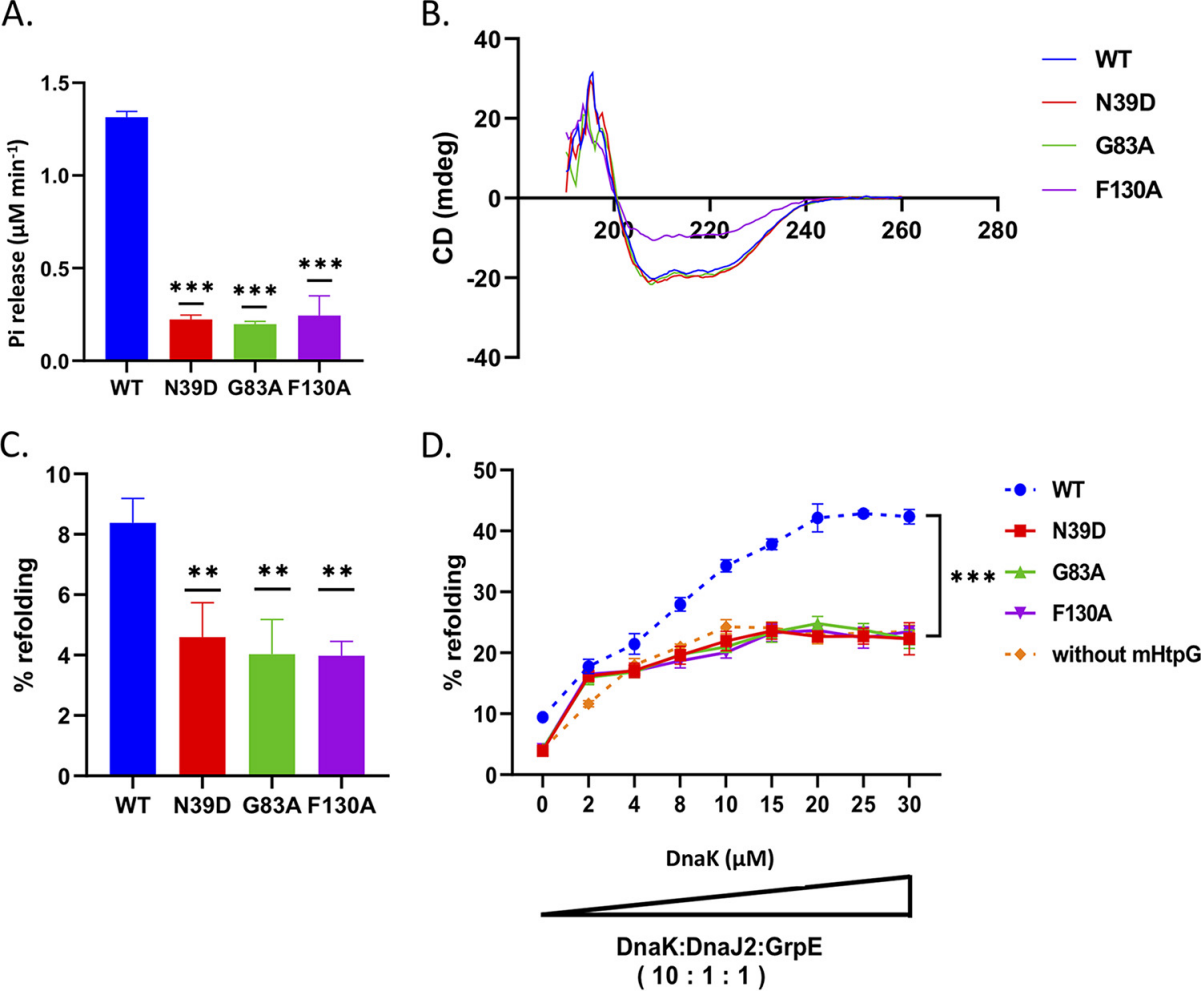
ATPase activity is critical for the refolding activity of mHtpG. (A) Effect of single amino acid substitutions on ATPase activity of mHtpG. ATP hydrolyzing activity was investigated in wild-type and substitution mutants of mHtpG to understand the importance of specific amino acid residues, which reveals that all the N39D, G83A, and F130A residues are critical for its ATPase activity. (B) Analysis of the conformation of mutant mHtpG proteins. CD spectroscopy was performed to analyze the structural information of WT mHtpG and other substitution mutants, which reveals no effect on protein folding due to these substitutions. (C and D) Effect of mutant mHtpG on protein refolding, either alone (C) or with KJE (D). A luciferase refolding assay was performed using WT, N39D, G83A, and F130A mHtpG proteins (2 μM each) with 80 nM heat-denatured luciferase, which shows an ~50% reduction in the protein refolding due to any of these substitutions (C). Similarly, none of these mutant mHtpG variants are able to enhance the refolding activity of the KJE chaperone, indicating the importance of ATPase activity in mHtpG for protein refolding. The broken lines in panel D represent data presented in Fig. 3C that are used for reference purpose. All the reactions in panels (A, C, and D) were performed in triplicate, and the mean ± SD values are shown. Asterisks represent the level of significance, as determined by Student’s *t* test. **, *P* < 0.005; ***, *P* ≤ 0.0005.

Next, we examined whether ATP hydrolyzing activity of mHtpG is critical for its effect on DnaK. Independent substitutions of the ATP binding residues in mHtpG at three randomly selected positions *viz.*, N39D, G83A, and F130A (see Fig. S3 at https://thsti.in/Supplementary%20Information_Mangla%20et%20al%20Final-R1.pdf), were made by site-directed mutagenesis, and the respective mutant proteins were purified as described in Materials and Methods. It was observed that these substitutions led to a drastic reduction in the ATPase activity by ~80 to 85% compared to wild-type protein (Fig. 4A). Analysis of the secondary structure of these by circular dichroism (CD) spectroscopy confirms that this defect in ATP hydrolysis is not an outcome of structural deformity owing to N39D, G83A, and F130A substitutions (Fig. 4B), and is probably due to a lack of ATP recognition by these mutant proteins. It was observed that mutation at these sites results in a reduction in the refolding activity to nearly half that of the wild-type mHtpG (Fig. 4C). Next, to check the consequence of defective ATPase activity exhibited by mHtpG mutant proteins on chaperonin activity of KJE, we determined the luciferase refolding activity of KJE in the presence of these mHtpG variants. Our results show that any of these substitutions cause complete loss of stimulation of KJE chaperonin activity (Fig. 4D). Taken together, these results indicate that ATPase activity of mHtpG is obligatory for its chaperone function and subsequent activation of KJE complex.

### mHtpG directly associates with the KJE chaperone system via DnaJ2-mediated physical interaction.

**FIG 5:**
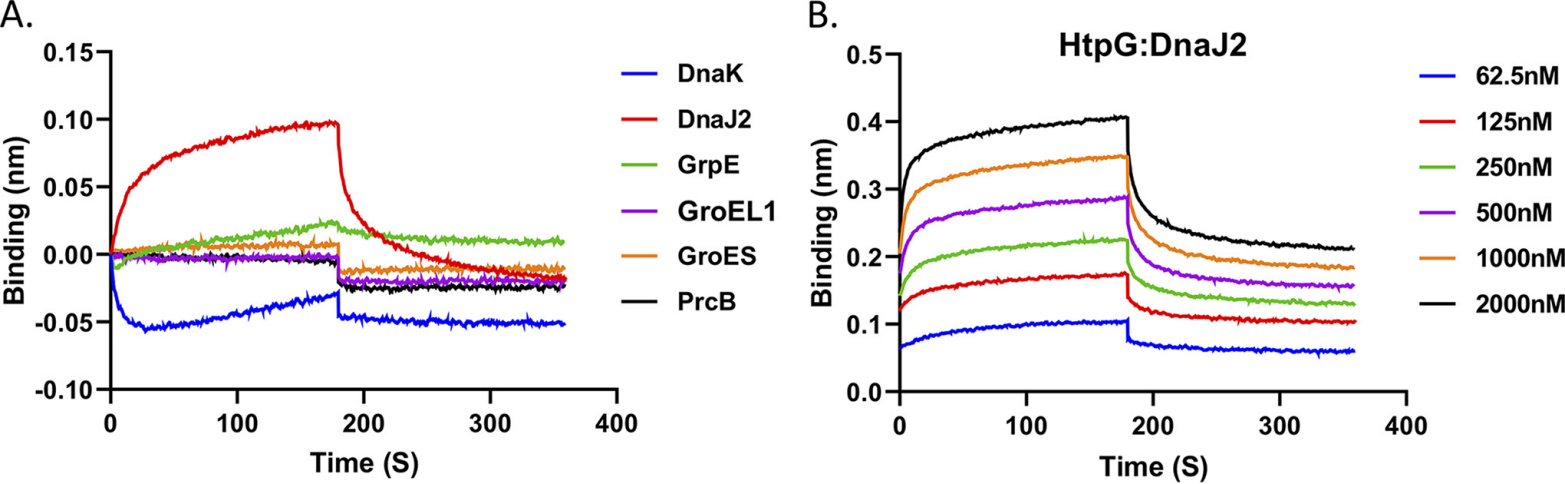
Analysis of the interaction of mHtpG with other mycobacterial chaperones. (A) Interaction analysis between mHtpG and various chaperons. BLI-octet was used for analyzing the interaction between mHtpG and DnaK, DnaJ2, GrpE, GroEL1, GroES, and PrcB, as described in Materials and Methods. PrcB was used as a negative control. Based on the response, mHtpG appears to interact with only DnaJ2. (B) Analysis of binding kinetics of DnaJ2 with mHtpG. The interaction of mHtpG and DnaJ2 at different concentrations of DnaJ2 reveals strong binding with a dissociation constant (*K_d_*) of ~200 nM. The *K_d_* was estimated by using Octet software. Data are representative of two independent experiments in panels A and B.

To explore the mechanistic understanding of the collaborative function of the mHtpG and KJE chaperone systems, we next investigated the interacting partner of mHtpG in the KJE complex by using the bio-layer interferometry (BLI) approach. In other organisms Hsp90 is known to interact physically with Hsp70 (47, 48). As described in Materials and Methods, we used mHtpG as bait protein which was immobilized on the AR2G sensor, whereas various chaperonin components were used as prey to check their interaction with mHtpG. An unrelated protein, PrcB, of the proteasome machinery was used as a control. To our surprise, we were unable to observe any direct interaction between mHtpG and DnaK, unlike what is reported in other organisms. Likewise, no association could be established between mHtpG and GrpE by the BLI method. Importantly, the mHtpG is found to interact exclusively with DnaJ2 (Fig. 5A). Furthermore, no interaction is seen with other chaperons such as GroEL1 and GroES as well as PrcB, which together imply a specific effect of mHtpG on the KJE system of mycobacteria via direct association with DnaJ2. Next, we analyzed binding kinetics using various concentrations of DnaJ2 ranging from 62.5 to 2000 nM, which further reveals that mHtpG binds strongly with DnaJ2 with a dissociation constant (*K_d_*) of ~200 nM (Fig. 5B).

### Loss of *mhtpG* results in overabundance of DnaJ cochaperons and Clp unfoldases.

**FIG 6:**
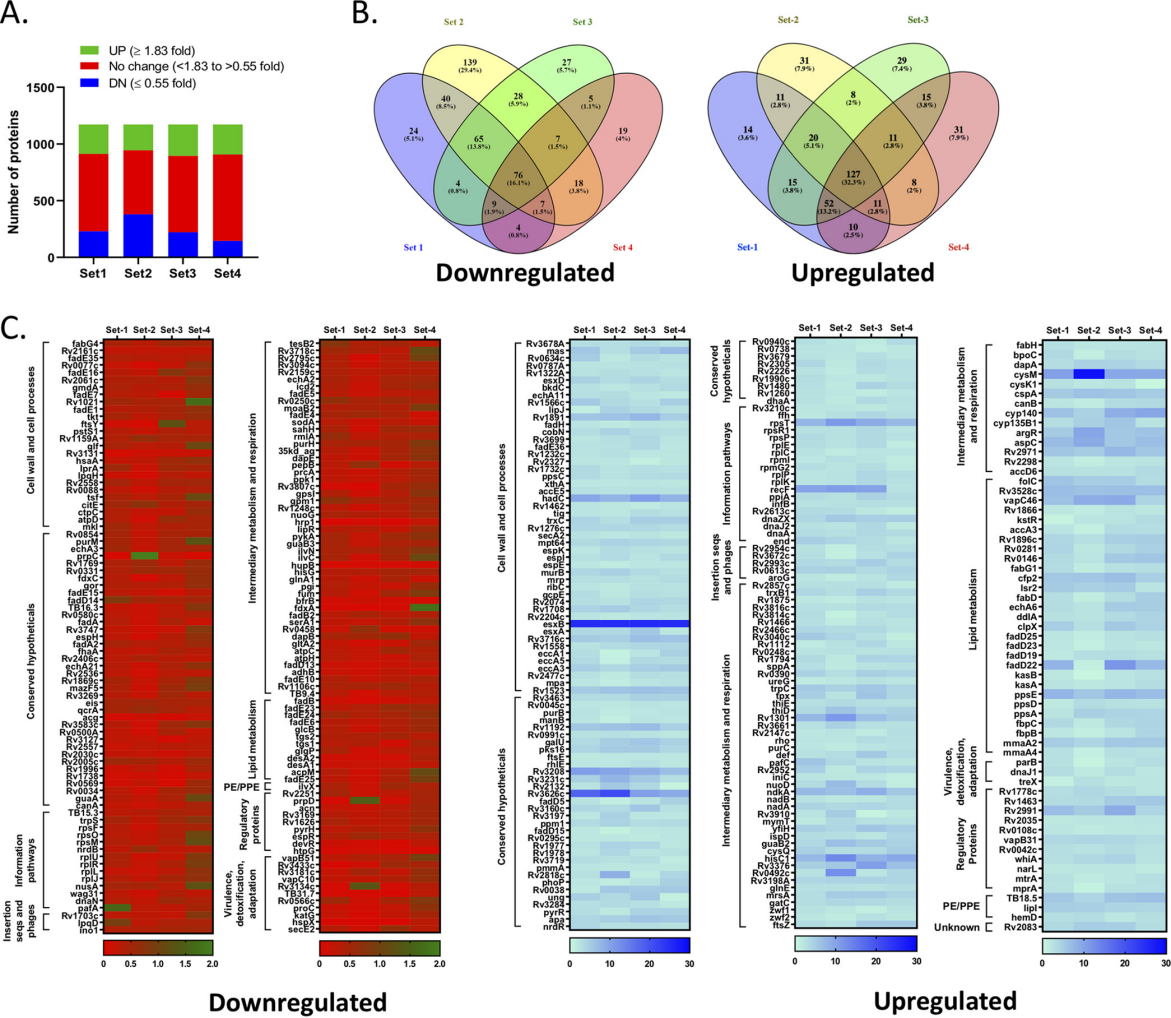
Quantitative proteomic analysis of Δ*htpG*. (A) Status of differentially regulated proteins in different biological replicates. The bar graph depicts downregulated (≤0.55-fold) (DN), unchanged (<1.83-fold to >0.55-fold) (no change), and upregulated (≥1.83-fold) (UP) proteins in the Δ*htpG* strain of MtbH37Rv across four biological replicates (set 1 to set 4). (B) Analysis of differentially expressed proteins across replicate samples. The upregulated and downregulated proteins in four biological replicates are represented by the Venn diagrams to identify consistency in the expression pattern. The Venn diagrams were generated by using Venny 2.1 software. (C) Heat map analysis of differentially regulated proteins. Heat maps show both down- and upregulated proteins classified under different functional categories. The results reveal that loss of HtpG in M. tuberculosis leads to the downregulation of 164 proteins and upregulation of 221 proteins in at least 3 of the 4 biological replicates, attributed with different functional categories.

Next, to comprehend the requirement of mHtpG for survival of M. tuberculosis, we generated an *mhtpG* deletion mutant strain with a phage-based technique (49), as described in Materials and Methods, and confirmed it by PCR based analysis (see Fig. S4A at https://thsti.in/Supplementary%20Information_Mangla%20et%20al%20Final-R1.pdf), Southern blotting (see Fig. S4B at the URL mentioned above), and anti-mHtpG immunoblotting (see Fig. S4C at the URL mentioned above). Analysis of *in vitro* growth of the wild type, Δ*htpG* mutant, and Δ*htpG*::*htpG* complemented strains in the synthetic broth medium reveals no difference in the growth profile of M. tuberculosis upon deletion of *mhtpG* (see Fig. S5 at https://thsti.in/Supplementary%20Information_Mangla%20et%20al%20Final-R1.pdf). Although mHtpG is maintained by M. tuberculosis throughout the course of genetic evolution, we have yet to understand how M. tuberculosis is able to endure the loss of this protein. Hence, to enlighten the pathogen’s response to the loss of mHtpG and to further understand its effect on bacterial physiology, quantitative proteomics by isobaric tags for relative and absolute quantitation (iTRAQ) was performed with the whole-cell lysates (WCLs) of the wild-type and Δ*htpG* strains of M. tuberculosis H37Rv, typically as described previously (50, 51). We adopted an 8-plex iTRAQ-based quantitative proteomics approach to identify proteins that exhibit a consistent pattern of expression across 4 biological replicates (Fig. 6A). A total of 1,172 proteins, identified by ≥2 peptides, were considered (see Data set S1 at https://thsti.in/Suppl.%20Dataset%201.xlsx) to determine the fold change in expression levels of proteins in the mutant compared to the control in all the replicates. Proteins exhibiting a ≤0.55-fold reduction (*P* ≤ 0.05) in expression were considered downregulated, whereas those with ≥1.83-fold (*P* ≤ 0.05) overexpression were considered upregulated (see Data Sets 2 and 3 at https://thsti.in/Suppl.%20Dataset%202.xlsx and https://thsti.in/Suppl.%20Dataset%203.xlsx, respectively). Analysis of these proteins further reveals that 76 proteins are downregulated and 127 are upregulated in Δ*htpG* compared with the control across all four biological replicates (Fig. 6B). In addition to these, 88 proteins exhibit downregulation and 94 exhibit upregulation in any 3 of the 4 biological replicates (Fig. 6B). Overall, these results signify the physiological impact of loss of HtpG on the global expression pattern of M. tuberculosis proteins. To gain an insight into the function of differentially regulated proteins, these were classified into various functional categories as predicted in the Mycobrowser database (https://mycobrowser.epfl.ch/). Of the total 385 proteins showing altered expression in the mutant, 29% belong to intermediary metabolism and respiration, 21% to conserved hypotheticals, 19% to cell wall and cell processes, 11% to lipid metabolism, and 9% to information pathways, and 5% are regulatory proteins and 4% are involved in virulence, detoxification, and adaptation. As can be seen in Fig. 6C, a vast majority of proteins involved in different metabolic activities such as cell division, cell wall biosynthesis, DNA transcription, replication and translation, and energy metabolism are modulated in the Δ*htpG* mutant strain. Notably, absence of mHtpG does not influence other major chaperones such as DnaK, GroEL, and GroES. However, we find increased expression of DnaJ1 and DnaJ2 cochaperones of the KJE system which exhibit 2.44- and 2.58-fold upregulation, respectively, in the Δ*htpG* strain. Other than the protein refolding system, we also find modulation in the expression of caseinolytic proteolytic machinery components, ClpC1 and ClpX unfoldases, which are upregulated by 2.00- and 4.32-fold, respectively, in the mutant strain (Fig. 6C). Upregulation of ClpC1 in the Δ*htpG* strain was also verified by anti-ClpC1 immunoblotting, which shows an ~1.7-fold increase relative to its level in the wild-type control and ~1.4-fold compared with the complemented strain (see Fig. S6 at https://thsti.in/Supplementary%20Information_Mangla%20et%20al%20Final-R1.pdf).

## DISCUSSION

Proteostasis is one of the fundamental cellular processes and is essential for the vitality of all kinds of living cells. Chaperones play a major role in the maintenance of proteostasis. Although HtpG, also known as Hsp90, is not essential for *in vitro* growth of E. coli (29), it is required for the CRISPR-mediated immunity against λ prophage (52). HtpG plays an important role in maintaining functional levels of Cas3, a protein which is essential for the CRISPR system of E. coli (52). It is also required for virulence of the pathogenic E. coli wherein it regulates the synthesis of the toxin colibactin and the siderophore yersiniabactin by modulating the ClpQ protease (39). In Salmonella enterica serovar Typhimurium, loss of HtpG results in compromised motility, reduction in biofilm, poor adhesion, and invasion of *S.* Typhimurium, thus affecting its inflammation-inducing ability (53).

Although HtpG is conserved in M. tuberculosis complex (MTBC) bacteria, functional characterization of this protein from M. tuberculosis remains pending. While the presence of Mg^++^ is a strict requirement for the ATPase activity of HtpG chaperones, including mHtpG (Fig. 2A), interestingly, mHtpG shows relatively better activity in the presence of Ca^++^ ions, with a >2-fold increase in affinity and ~1.5-fold increase in the rate of hydrolysis of ATP compared to what was observed in the presence of Mg^++^. Importantly, Mg^++^ could not be substituted with any other divalent cations, suggesting a strict dependency of this protein on Mg^++^ and Ca^++^ for its ATP hydrolysis activity (Fig. 2). A similar finding has been reported in a previous study wherein it was observed that Ca^++^ can replace Mg^++^, the essential enzymatic cofactor, to support TRAP1 (mitochondrial HSP90) ATPase activity (54). The maximum activity of mHtpG was observed at a high temperature of 50°C, thus justifying it being a high-temperature chaperone (Fig. 2B and C).

We have observed that mHtpG alone exhibits a mild refolding activity with the denatured model substrate which requires ATP hydrolysis. Mutation of all the three residues, *viz.*, N39D, G83A, and F130A, which are critical for ATP binding, led to an ~50% reduction in the refolding of the denatured substrate (Fig. 4C). These results further indicate that although the presence of ATP is crucial, it is not a strict requirement for substrate refolding by mHtpG, which is in accordance with its counterpart from other organism (55).

**FIG 7:**
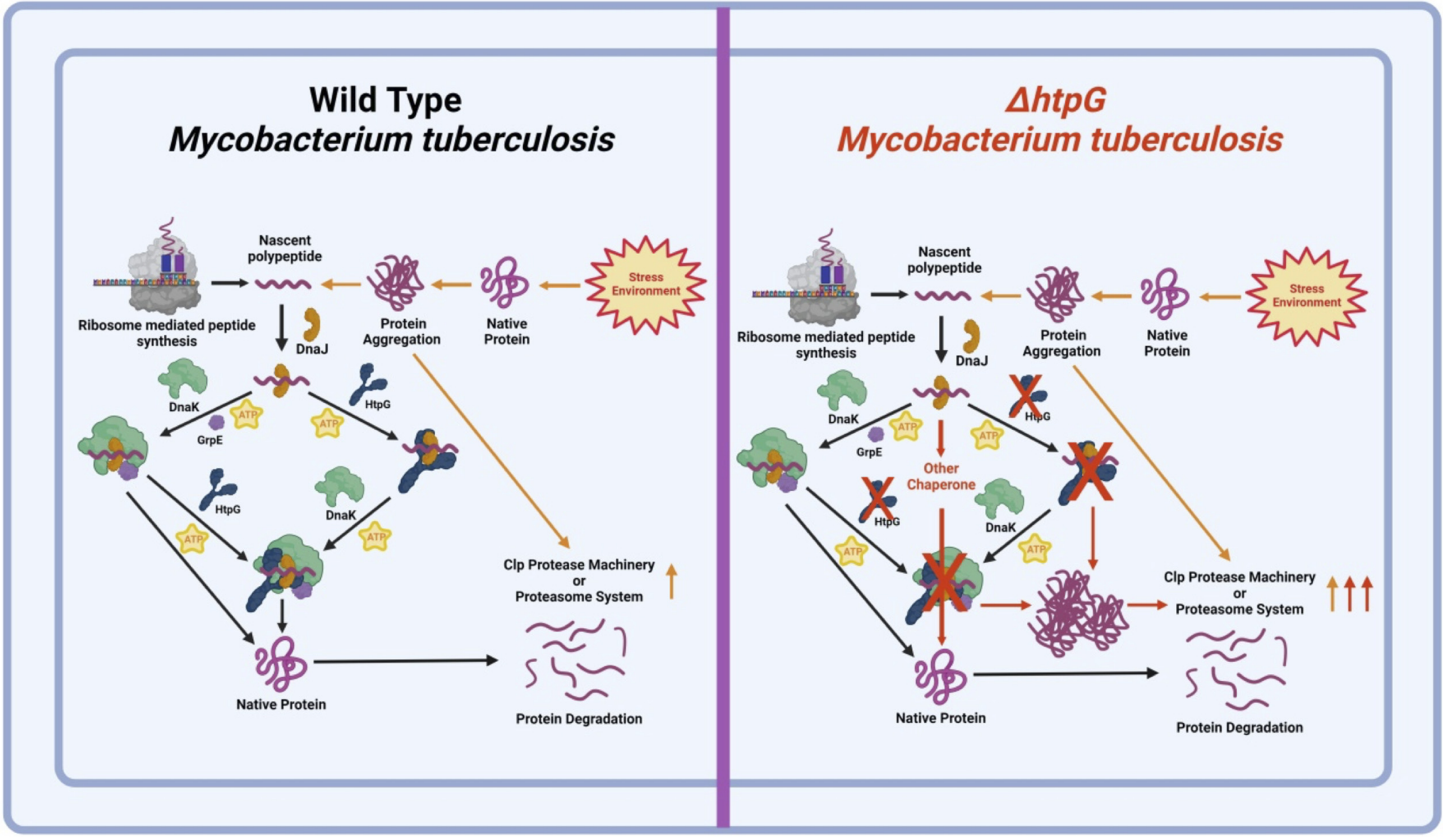
Graphical representation depicting maintenance of proteostasis in the wild-type and Δ*htpG* strains of M. tuberculosis. In the wild-type cells, the newly synthesized protein (left) or the stress-induced protein aggregate (right) is recognized by the cochaperonin, DnaJ, which is transferred to the associated mHtpG or to some other chaperones. The mHtpG subsequently causes partial refolding of the substrate in an ATP-dependent manner, which is then passed on to DnaK in the presence of GrpE, resulting in proper folding. In the Δ*htpG* strain, expression of DnaJ is enhanced to compensate for the loss of mHtpG, leading to direct transfer of the candidate protein to the DnaK chaperone, thus maintaining the homeostasis. Simultaneously, a fraction of protein, which remains in the aggregated form or which is unable to be folded properly, is taken care of by major protease machinery of the cell, such as ClpX or ClpC1-associated proteases, which are activated in the absence of mHtpG.

The KJE complex constitutes the primary chaperone machinery in the cell, which is essential for protecting proteins from heat-induced aggregation (46, 56, 57). Importantly, the KJE chaperon machinery receives assistance from several other chaperons, including Hsp90 (58–60). In light of this information and owing to weak refolding activity of mHtpG, it was further investigated whether mHtpG affects the refolding activity of the mycobacterial KJE chaperone. Remarkably, the presence of mHtpG causes a 100% increase in the refolding of denatured substrate by KJE (Fig. 3C), which is strictly dependent on the ATPase activity of mHtpG (Fig. 4D). Since the KJE chaperon exhibits a strong affinity with the denatured substrate proteins (44), our results indicate that there is a likely effect of mHtpG on the refolding activity of KJE, and not on its substrate affinity. Furthermore, using protein-protein interaction studies, we show that the effect of mHtpG on KJE is mediated through its direct interaction with the chaperone via DnaJ2. These observations put forth a possible mechanism for the substrate refolding by the mHtpG-KJE complex in M. tuberculosis, wherein nonnative substrate protein is approached by DnaJ2/DnaJ1 cochaperones, which further direct it to mHtpG associated with DnaJ2. Subsequently, the mHtpG induces partial refolding of the substrate protein and primes it for subsequent refolding by the DnaK chaperone in an ATP-dependent manner (Fig. 7). A similar effect has been observed with the eukaryotic HSP70 system, which collaborates with HSP90 and a few other cochaperones such as Sti1/Hop for reactivation of the denatured luciferase (47, 60–62). Earlier *in vitro* studies indicate that HSP70 first recognizes denatured luciferase, which is then transferred to HSP82 (Hsp90 homolog) via its connection with Sti1 (61), which bridges HSP82 and HSP70 during this process. Although refolding of denatured proteins by the mHtpG and KJE systems *in vitro* takes place in the absence of any other proteins, involvement of other cochaperone(s) in this process *in vivo* cannot be ruled out and would require further investigation.

Whole-cell proteomic analysis of Δ*htpG* provides an important insight into the mechanism by which the mutant cells maintain cellular homeostasis by upregulating the DnaJ1 and DnaJ2 cochaperones along with Clp unfoldases, ClpC1 and ClpX. The overabundance of these proteins appears to be a compensatory response of the pathogen to either limit the accumulation of protein aggregates or to expedite their clearance by Clp proteolytic machinery, which also explains the redundant requirement of mHtpG in the TB pthogen. Intrigued with these observations, we propose further study to analyze the effect of depletion of some or all of these chaperones in the Δ*htpG* strain on bacterial growth and physiology, which will shed an important light on possible cross talk among various chaperons, including mHtpG, *in vivo*.

## MATERIALS AND METHODS

### Plasmids, bacterial strains, and culture conditions.

All the plasmids and bacterial strains used in this study are listed in Table 1. E. coli DH5α and BL21 (λDE3) strains were used for cloning and protein purification and were grown in Luria-Bertani (LB) broth or on agar medium (Becton, Dickinson Difco, USA). Mycobacterium tuberculosis H37Rv and Mycobacterium smegmatis mc^2^155 strains were cultured in Middlebrook 7H9 broth or 7H11 agar (Becton, Dickinson Difco, USA) containing 10% (vol/vol) OADS (oleic acid-albumin-dextrose-saline) and 0.5% (vol/vol) glycerol. Middlebrook 7H9 broth was also supplemented with 0.05% (vol/vol) tyloxapol (Sigma-Aldrich, USA). Bacteria were cultured at 37°C, with or without shaking at 180 rpm, unless otherwise mentioned. We used 100 μg/mL ampicillin, 50 μg/mL kanamycin, and 100 μg/mL hygromycin for recombinant E. coli strains and 25 μg/mL kanamycin and 50 μg/mL hygromycin for culturing of recombinant mycobacterial strains, as per the requirements.

**TABLE 1:** Bacterial strains and plasmids used in this study

Strain or plasmid	Antibiotic resistance	Source or reference
Bacterial strains		
E. coli DH5α		Thermo Fisher Scientific, USA
E. coli strains BL21 (λDE3)		Thermo Fisher Scientific, USA
M. tuberculosis H37Rv		Kindly provided by Dr. William Bishai, Johns Hopkins University, USA
M. smegmatis mc²155		Kindly provided by Dr. William Jacobs, Albert Einstein College of Medicine, USA
M. tuberculosis H37Rv Δ*htpG*	Hygromycin	This study
M. tuberculosis H37Rv Δ*htpG*::*htpG*	Kanamycin and hygromycin	This study
Plasmids		
pGEX-6P-1	Ampicillin	Sigma Aldrich, USA
pGEX-6P-1_HtpG	Ampicillin	This study
pGEX-6P-1_DnaJ2	Ampicillin	This study
pGEX-6P-1_HtpG N39D	Ampicillin	This study
pGEX-6P-1_HtpG G83A	Ampicillin	This study
pGEX-6P-1_HtpG F126A	Ampicillin	This study
pGEX-6P-1_HtpG G127A	Ampicillin	This study
pGEX-6P-1_HtpG F130A	Ampicillin	This study
pET28	Kanamycin	Novagen, USA
pET28_DnaK	Kanamycin	This study
pET28_GrpE	Kanamycin	This study
pET28_GroEL1	Kanamycin	This study
pET28_GroES	Kanamycin	This study
pET28_PrcB	Kanamycin	This study
pYUB854	Hygromycin	49; obtained from Dr. William Jacobs, Albert Einstein College of Medicine, USA
phAE159		49; obtained from Dr. William Jacobs, Albert Einstein College of Medicine, USA

### Cloning, expression, and purification of proteins.

The *htpG* (Rv2299c), *dnaK* (Rv0350), *dnaJ2* (Rv2373c), *grpE* (Rv0351), *groES* (Rv3418c), and *prcB* (Rv2110c) genes were PCR amplified using gene-specific oligonucleotides as mentioned in Table 2 from M. tuberculosis H37Rv genomic DNA. The *htpG* and *dnaJ2* genes were cloned in a modified version of an E. coli expression vector, pGEX-6P-1, harboring NdeI and HindIII restriction endonuclease sites, whereas the *grpE*, *groES*, and *prcB* genes were cloned in pET28a vector at the NdeI and HindIII restriction sites. The PCR amplicon of *dnaK* was cloned in a TA cloning vector, pTZ57R/T (Thermo Fisher Scientific, USA), and the fragment of ~1.9 kb was obtained by NdeI and HindIII digestion and cloned at the same sites in pET28a vector. These clones were prepared such that the respective recombinant proteins carry GST- or 6× His tag at their N terminus, respectively. The pET28c-groEL1 was obtained from Rajesh Gokhale, Institute of Genomics and Integrative Biology, India. E. coli DH5α was used for propagation of the recombinant clones that were confirmed by DNA sequencing before proceeding to the protein expression and purification. Plasmid DNAs were subsequently transformed into an expression host, E. coli BL21 (λDE3), for protein expression. Transformants were selected in the presence of the respective antibiotics on LB agar plates after overnight incubation at 37°C. A single bacterial colony was picked for inoculation of the primary culture in 10 mL LB broth containing antibiotic and allowed to grow overnight with shaking. Secondary culture was inoculated in 500 mL LB broth containing antibiotic and grown to an optical density at 600 nm (OD_600_) of 0.6 to 0.8. Protein expression was induced by the addition of 1 mM IPTG (isopropyl-β-d-thiogalactopyranoside) for 16 h at 18°C. Afterward, bacterial culture was centrifuged at 8,000 rpm for 10 min, and the pellet was washed and suspended in the lysis buffer (50 mM Tris-HCl [pH 8.0], 150 mM NaCl, and 10% glycerol). Cells were lysed in lysis buffer containing 1 mM phenylmethylsulfonyl fluoride (PMSF; Sigma-Aldrich, USA) with the help of PandaPLUS laboratory homogenizer. WCLs were then centrifuged at 12,000 rpm for 15 min at 4°C to remove pellet containing cell debris. Clarified lysates were allowed to bind with glutathione-sepharose or Ni-NTA resin (Thermo Fisher Scientific, USA) for 16 to 18 h at 4°C with mixing for immobilization of GST- or His-6×-tagged proteins on the respective resins. The GST-tagged mHtpG and DnaJ2 proteins were eluted by incubation of the resin with 20 mM reduced glutathione in the lysis buffer, followed by removal of GST-tag by overnight incubation with PreScission protease (Cytiva, Merck, USA) in the lysis buffer containing 1 mM dithiothreitol (DTT) and 1 mM EDTA. The His-tagged proteins were eluted from the resin by using 100-300 mM imidazole in the lysis buffer. The purity of the proteins was analyzed with SDS-PAGE through Coomassie brilliant blue staining. Fractions with >95% purity were pooled and stored in multiple aliquots at −80°C for subsequent use.

**TABLE 2:** Oligonucleotides used in this study

Primers	Sequence (5′-3′)	Remark^*a*^
Pr. 659	NNNCATATGAACGCCCATGTCGAGCAGTTGG	Forward primer for PCR amplification of *mhtpG* ORF
Pr. 660	NNNAAGCTTCTACAAGGTACGCGCGAGACG	Reverse primer for PCR amplification of *htpG* ORF
Pr. 1086	NNTCTAGAGCCAAACCCCTCGATTCCATC	Forward primer for PCR amplification of *htpG* ORF with 5′ UTR
Pr. 1088	NNGGATCCGAGATTCCTTCCGACGACATAG	Reverse primer for construction of labeled probe for Southern hybridization
Pr. 836	GTTGATCTCGGATGCCTCCGATG	Forward primer for PCR amplification of *htpG* ORF with N39D mutation
Pr. 837	CATCGGAGGCATCCGAGATCAAC	Reverse primer for PCR amplification of *htpG* ORF with N39D mutation
Pr. 838	CGTGACAACGCCATCGGGATG	Forward primer for PCR amplification of *htpG* ORF with G83A mutation
Pr. 839	CATCCCGATGGCGTTGTCACG	Reverse primer for PCR amplification of *htpG* ORF with G83A mutation
Pr. 844	CGGCATCGGCGCCTACTCGTCG	Forward primer for PCR amplification of *htpG* ORF with F130A mutation
Pr. 845	CGACGAGTAGGCGCCGATGCCG	Reverse primer for PCR amplification of *htpG* ORF with F130A mutation
Pr. 103	GCGAGGTGGACTTGAGTTCC	Forward primer for assessment of *htpG* deletion
Pr. 104	GTGTCATCCCGAAGGCATCG	Reverse primer for assessment of *htpG* deletion
*dnaK*(F)	CCCCCATATGGCTCGTGCGGTCGGGAT	Forward primer for PCR amplification of *dnaK* ORF
*dnaK*(R)	GGTTAATTAATCACTTGGCCTCCCGGCCGTCG	Reverse primer for PCR amplification of *dnaK* ORF
Pr. 1012	NNCATATGGCACGCGATTATTACGGG	Forward primer for PCR amplification of *dnaJ2* ORF
Pr. 1013	NNAAGCTTAGCGCCCGGTGAAGGTCTC	Reverse primer for PCR amplification of *dnaJ2* ORF
Pr. 848	NNCATATGACGGACGGAAATCAAAAGCCGG	Forward primer for PCR amplification of *grpE* ORF
Pr. 849	NNAAGCTTAACTGCCCGACGGTTCTGATTCTGCC	Reverse primer for PCR amplification of *grpE* ORF
Pr. 1089	NNCATATGGCGAAGGTGAACATCAAGC	Forward primer for PCR amplification of *groES* ORF
Pr. 1090	NNAAGCTTCTACTTGGAAACGACGGCCAGC	Reverse primer for PCR amplification of *groES* ORF
Pr. 852	NNCATATGACCTGGCCGTTGCCCGATC	Forward primer for PCR amplification of *prcB* ORF
Pr. 853	NNAAGCTTCACTTCTCACCGCCATCGGAGCC	Reverse primer for PCR amplification of *prcB* ORF
*htpG* KO _UP_FP	GAGGCCTATGCCACGTTCTGCTCGTCGAGACCG	Forward primer for cloning of the left flank of *htpG* in pYUB854
*htpG* KO _UP_RP	GTCTAGAAAACTCCAACTGCTCGACATGGGCGTTC	Reverse primer for cloning of the left flank of *htpG* in pYUB854
*htpG* KO _DN_FP	GAAGCTTCTGGCCGAACGTCTCGCGCGTACC	Forward primer for cloning of the right flank of *htpG* in pYUB854
*htpG* KO _DN_RP	GACTAGTGGCCTGGGCAAAAGCGAGCGTATTC	Reverse primer for cloning of the right flank of *htpG* in pYUB854

a ORF, open reading frame.

After cleavage of the tag, mHtpG protein was purified by size exclusion chromatography using a Superdex S-200 Increase 10/300 column on an AKTA fast protein liquid chromatography (FPLC) system (Cytiva, Merck, USA), as per the manufacturer’s recommendation.

### Site-directed mutagenesis.

Point mutant derivatives of M. tuberculosis *htpG* were generated using a QuickChange site-directed mutagenesis kit (Agilent, USA). Briefly, forward and reverse primers carrying mutations (Table 2) were used for incorporation of mutation in *htpG* using pGEX-6p-1_htpG as the template, typically as described by the manufacturer. The wild-type template was destroyed by incubation with 1 μL of the DpnI enzyme at 37°C for 1 h, followed by precipitation of DNA with ethanol. DNA samples were transformed into E. coli DH5α, and clones were screened for incorporation of the respective mutations by sequencing. All the mutant proteins, N39D, G83A, and F130A, were subsequently purified as described above.

### Determination of ATPase activity in mHtpG.

ATPase activity of purified mHtpG was determined using a malachite green phosphate assay, as described previously (63). A typical ATPase assay was performed with 1 μM protein in a 1× ATPase assay buffer (20 mM HEPES-KOH, pH 7.5, 100 mM KCl, 36.7% glycerol, and 10 mM MgCl_2_) at 37°C for 30 min followed by estimation of released inorganic phosphate by measuring the absorbance at 630 nm. To check the effect of different divalent cations, MgCl_2_ in the reaction buffer was replaced with CaCl_2_, MnCl_2_, ZnCl_2_, CdCl_2_, CoCl_2_, NiCl_2_, BaCl_2_, or CuSo_4_. Reactions set at different temperatures and various pHs of the buffer were used to identify the optimum conditions for achieving the maximum ATPase activity of mHtpG. To determine the kinetics of ATP hydrolysis, reactions were performed in the assay buffer containing various concentrations of ATP.

### Determination of refolding activity in mHtpG.

Refolding activity in mHtpG was determined in the presence or absence of M. tuberculosis KJE complex using firefly luciferase (Sigma-Aldrich, USA) as a model substrate. Luciferase was denatured by heat treatment for 10 min at 45°C such that ~98% of its luminescence was lost. The denatured protein was then incubated with purified chaperone proteins at different concentrations, as described in the text, in a refolding buffer (25 mM HEPES-KOH, pH 7.5, 50 mM KCl, 15 mM MgCl_2_, and 50 μg/mL bovine serum albumin [BSA]) containing 2.5 mM ATP, 2 mM DTT, 2 mM creatine phosphate, and 0.25 mg/mL creatine phosphokinase. The reaction was incubated at 25°C for 30 min to induce refolding of firefly luciferase to its active conformation. Luminescence in the refolded luciferase was subsequently measured in a luminometer after addition of luciferin (Sigma-Aldrich). Native luciferase was used as a positive control to measure the extent of refolding in the heat-denatured luciferase by chaperones.

### Protein-protein interaction analysis.

The interaction of mHtpG with different proteins was analyzed by BLI-octet, as per the instructions by the manufacturer (Sartorius, Göttingen, Germany). Briefly, mHtpG protein was immobilized onto the AR2G sensor (second generation) after dialysis in 10 mM sodium acetate, pH 3.5. Interaction was performed using 5 μM purified proteins in a buffer containing 50 mM Tris-HCl, pH 8.0, and 150 mM NaCl. Binding was analyzed after completing the standard steps of baseline (60 s), association (180 s), and dissociation (180 s). The baseline was set using analyte buffer as a control. Different concentrations of purified DnaJ2 were used to acquire a differential graded response for calculation of the binding constant (*K_d_*).

### Construction of Δ*htpG* and Δ*htpG*::*htpG* M. tuberculosis H37Rv stains.

Deletion of *htpG* in M. tuberculosis H37Rv was performed by a specialized transduction approach, as described earlier (49). Briefly, 800 bp of upstream and downstream regions flanking *htpG* were amplified by PCR using specific primers (Table 2) and cloned into cosmid vector pYUB854 flanking the hygromycin resistance gene at the StuI-XbaI and HindIII-SpeI sites, respectively. The recombinant cosmid was subsequently packaged into phAE159 using Gigapack III packaging extract (Agilent, USA). The recombinant phagemid was electroporated in M. smegmatis to generate a high-titer temperature-sensitive mycobacteriophage. For construction of the mutant strain, mid-log-phase culture of M. tuberculosis was transduced with the mycobacteriophage, and transductant was selected on 7H11 agar plates containing hygromycin. Deletion of *htpG* from the genome of the prospective mutant strain was initially assessed by PCR using gene-specific primers (primer [pr.] 103 and pr. 104, Table 2) (see Fig. S4A at the URL mentioned above) (50) and then confirmed by Southern blotting with the help of labeled probe (see Fig. S4B at the URL mentioned above). Absence of mHtpG expression in the mutant strain was confirmed by Western blotting using anti-mHtpG antibodies (see Fig. S4C at the URL mentioned above), as described previously (50).

For construction of the Δ*htpG*::*htpG* complemented strain of M. tuberculosis H37Rv, the *htpG* coding sequence along with a 400-bp upstream region was PCR amplified using pr. 1086 and pr. 660 (Table 2), restriction digested with XbaI and HindIII, and cloned in a Kan^R^ integrative plasmid, pTetInt (64), at the XbaI and HindIII restriction sites replacing the entire TetR cassette and Tet-inducible promoter. The resulting plasmid, pInt-htpG was subsequently electroporated in the Δ*htpG* strain, and restoration of *htpG* expression was confirmed in the complemented strain by anti-HtpG immunoblotting (see Fig. S4C at the URL mentioned above).

### Southern blotting.

Genomic DNAs were extracted from all the three strains of M. tuberculosis H37Rv as described previously (65). Equal amounts of genomic DNA samples were digested with NotI enzyme and resolved on 1.2% agarose gel. An EtBr-stained gel image was captured under UV transilluminator, and the positions were marked for alignment. The gel was treated with depurination solution (0.2 N HCl) for 10 min, denaturation solution for 45 min (1.5 M NaCl and 0.5 M NaOH), and neutralization solution (1 M ammonium acetate) for 45 min. The gel was washed using autoclaved MilliQ (AMQ) water for 10 min after each of these treatments. Gel was then transferred to nylon membrane using a TurboBlotter kit (Whatman) in 20× SSC buffer (0.3 M sodium citrate and 3 M NaCl) overnight. The blot was air-dried, and DNA was cross-linked on the membrane by UV cross-linker for 2 min at 120 mJ/cm^2^. The North2South biotin random prime labeling kit (Thermo Fisher Scientific, USA) was used to generate biotin-labeled probe using pr. 1086 and pr. 1088. The North2South chemiluminescent hybridization and detection kit (Thermo Fisher Scientific, USA) was utilized for hybridization of the probe and signal detection according to the manufacturer’s instructions.

### Data availability.

The mass spectrometry proteomics data have been deposited at the ProteomeXchange Consortium via the PRIDE (66) partner repository with the data set identifier PXD039393.
